# Circulating serum preptin levels in women with polycystic ovary syndrome: A systematic review and meta-analysis

**DOI:** 10.18502/ijrm.v21i5.13470

**Published:** 2023-05-12

**Authors:** Seyed Sobhan Bahreiny, Elnaz Harooni, Mohammad Reza Dabbagh, Reza Ebrahimi

**Affiliations:** ^1^Student Research Committee, Ahvaz Jundishapur University of Medical Sciences, Ahvaz, Iran.; ^2^Department of Biology, Faculty of Sciences, Shahid Chamran University of Ahvaz, Ahvaz, Iran.; ^3^Department of Cell and Molecular Biology, Faculty of Chemistry, University of Kashan, Kashan, Iran.

**Keywords:** Polycystic ovary syndrome, Proinsulin-like growth factor II, Preptin, Meta-analysis.

## Abstract

**Background:**

Polycystic ovary syndrome (PCOS) is a prevalent endocrine disorder with complex pathogenesis and metabolic complications, such as insulin resistance. Among the new markers, preptin seems to play a significant role in metabolic disorders.

**Objective:**

This meta-analysis was conducted to determine the relationship between circulating preptin levels and PCOS.

**Materials and Methods:**

A systematic review and meta-analysis was performed to identify relevant articles in electronic databases such as PubMed, Web of Science, Scopus, Cochrane, EMBASE, and the Google Scholar search engine, using a predefined search strategy. A random-effects model was used to combine standard mean difference (SMD) and 95% CI to compare results between groups. Meta-regression and subgroup analysis were also performed to reveal the sources of heterogeneity.

**Results:**

The meta-analysis encompassed a total of 8 studies and 582 participants. The results indicate a statistically significant association between PCOS and serum preptin levels, with a pooled standardized mean difference (SMD = 1.35; 95% CI]: 0.63-2.08; p < 0.001). Further analysis suggested a significant difference in serum preptin levels between women with PCOS and higher homeostatic model assessment for insulin resistance ratio (SMD = 2.40; 95% CI: 1.17-3.63; p < 0.001) within the subgroup.

**Conclusion:**

Our meta-analysis shows that increased serum preptin levels are associated with PCOS, suggesting that preptin may be related to the pathogenesis of PCOS and may be recognized as a novel diagnostic biomarker for PCOS. However, further studies are needed to confirm our results.

## 1. Introduction

Polycystic ovary syndrome (PCOS) is a prevalent endocrine and metabolic disease in women of gestational age (1). It is characterized by ovulation disorder, hyperandrogenism, and polycystic ovarian morphology (2). Women with PCOS may suffer from various problems, such as infertility, endocrine, and metabolic disorders (3). The risk of depression and its symptoms appears to be higher in women with PCOS, which may be related to factors such as hyperinsulinemia, hyperandrogenism, and increased levels of inflammation (4, 5).

The underlying cause of PCOS is unknown and probably multifactorial, which has implications for proposed treatments. Many studies have suggested that PCOS is caused due to ovarian abnormalities (6–9). One criterion for PCOS is based on the Rotterdam criteria, which demands the presence of 3 features: hyperandrogenism, polycystic ovaries on ultrasound, and menstrual irregularities (10, 11). In addition, PCOS is closely associated with obesity, insulin resistance (IR), impaired glucose tolerance, hypertension, type 2 diabetes mellitus, dyslipidemia, and cardiovascular disease (12). Recent studies on metabolism-regulating proteins and peptides have gained interest as potential biomarkers for PCOS disorders (13–16).

Among the new markers involved in metabolic disorders, preptin is of great importance (17). Preptin is a 34-amino acids hormone produced by the gene encoding insulin-like growth factor 2. This peptide is co-secreted with insulin in response to glucose from the pancreatic beta-cells and stimulates insulin secretion (18, 19). The primary function of preptin involves the regulation of carbohydrate, protein, and lipid metabolism by modulating insulin secretion (20).

Studies show a direct relationship between preptin serum levels and the fasting homeostasis model assessment of insulin resistance (HOMA-IR) (21). Some studies have also showed the association between preptin serum levels and a high HOMA-IR in PCOS participants (18, 22). However, the effect of preptin on the pathogenesis of PCOS is still unclear, and the results seem controversial (23, 24).

Therefore, this meta-analysis aimed to analyze previous studies on the association between serum preptin levels and PCOS and to investigate whether preptin can be recommended as a new biomarker for PCOS.

## 2. Materials and Methods

### Protocol and registration

This systematic review and meta-analysis were conducted according to the preferred reporting items for systematic reviews and meta-analyses (PRISMA) guidelines to ensure transparency and rigor in the review process (25, 26). Our systematic review was registered in PROSPERO (the international database for prospectively registered systematic reviews).

### Eligibility criteria

All the observational studies published from January 2000 to August 2022 that defined PCOS according to the Rotterdam criteria and reported serum/plasma or follicular fluid preptin levels in PCOS participants compared with non-PCOS controls were included (10). In addition, some studies were excluded for the following reasons: I) incorrect comparison subjects and study design such as interventional studies, II) review articles, studies not conducted in humans, and conference articles, III) studies with non-extractable data.

### Literature search

A literature search was performed for human studies published in PubMed, Web of Science, Scopus, Cochrane, EMBASE, and Google Scholar search engines from January 2000 to August 2022. The following keywords were used: “Preptin”, “ProIGF-II”, “Insulin-Like Growth Factor II”, “polycystic ovary syndrome”, “PCOS”, “polycystic ovary” using coordinating conjunctions “AND” and “OR”. Also, references to related articles were searched manually. Search terms were supplemented by searching for available gray literature. In addition, all records were entered into the EndNote 9 software, and duplicate studies were removed after the screening.

### Study selection

2 independent reviewers thoroughly scanned the available literature to assess the eligibility criteria for the study (S.B. and M.D.). Any disagreements between the reviewers were resolved through collaborative discussion. In addition, a third reviewer was brought in to provide further insights and help reach a consensus (R.E.).

### Data extraction

The following data were extracted according to a pre-prepared checklist: origin of study characteristics (name of first author, year of publication, and location of study), study design (cross-sectional, cohort, or case-control), participant characteristics (age, number of participants, and body mass index), measurements, diagnostic criteria, and reported outcomes. In order to ensure the reliability and credibility of the evidence, the grading of recommendations assessment, development, and evaluation approach was applied to assess the quality of the studies included in this meta-analysis. Our evaluation indicated that the studies were between moderate and poor quality, mainly due to the risk of bias, the inconsistency of effect estimators, and imprecision caused by small sample sizes, leading to a high degree of uncertainty. Therefore, while our meta-analysis suggests a positive association between preptin levels and PCOS, caution should be exercised in interpreting the results. Nevertheless, our study adds to the existing body of knowledge on the role of preptin in the pathogenesis of PCOS and highlights the need for more research in this area (Table I).

**Table 1:** Characteristics of the studies included in the systematic review and meta-analysis


**Author, yr (Ref)**	**Country**	**Study designs**	**Number of participants**	**BMI (kg/m^2^) (PCOS vs. controls)**	**Age (yr) (PCOS vs. controls)**	**Sample (unit)**	**GRADE assessment**
**Mehmet ** ***et al.*** **,** **2022 (27)**	Turkey	Case-control	PCOS: 30 Control: 30	25.1 ± 2.01; 24.7 ± 4.08	28 (21-34); 27 (20-35)	Serum (pg/ml)	○ ⊕⊕⊕
**Ascar ** ***et al.*** **,** **2021 (28)**	Iraq	Case-control	PCOS: 50 Control: 50	N/A	28 (18-36); 28 (18-36)	Serum (pg/ml)	○○○ ⊕
**Ali ** ***et al.*** **,** **2018 (22)**	Iraq	Case-control	PCOS: 30 Control: 30	24.97 ± 2.23; 24.26 ± 3.53	26.73 ± 5.35; 29.38 ± 6.83	Serum (pg/ml)	○○ ⊕⊕
**Senturk ** ***et al.*** **,** **2018 (29)**	Turkey	Prospective randomized case-control	PCOS: 40 Control: 40	24.3 (19.1-52.8); 24.6 (17.7-34.9)	20.5 (17-35); 24 (17-35)	Serum (pg/ml)	○ ⊕⊕⊕
**Celik ** ***et al.*** **,** **2018 (30) **	Poland	Case-control	PCOS: 20 Control: 20	27.3 ± 4.90; 24.5 ± 4.96	31.0 ± 3.68; 35.3 ± 3.10	Serum, FF (ng/ml)	○ ⊕
**Mierzwicka ** ***et *** *al.*,**2018 (31)**	Turkey	Case-control	PCOS: 73 Control: 61	27.5 ± 6.4; 25.3 ± 4.7	24.3 ± 4.8; 29.2 ± 6.4	Serum (ng/ml)	○ ⊕⊕⊕
**Bu ** ***et al.*** **,** **2012 (32)**	China	Case-control	PCOS: 30 Control: 28	21.8 ± 2.7; 21.7 ± 3.2	26.1 ± 2.4; 28.4 ± 3.1	Serum (pg/ml)	○○ ⊕⊕
**Celik ** ***et al.*** **,** **2011 (17) **	Turkey	Cross-sectional	PCOS: 25 Control: 25	22.53 ± 5.31; 20.54 ± 1.95	23.52 ± 4.47; 23.12 ± 2.02	Serum (pg/ml)	○ ⊕⊕⊕
BMI: Body mass index, PCOS: Polycystic ovary syndrome, GRADE: Grading of recommendations assessment, development, and evaluation, FF: Follicular fluid. The rating of GRADE is as follows: ○ ⊕⊕⊕ Moderate quality: We are moderately confident about the effect estimate, ○○ ⊕⊕ Low quality: Our confidence in the effect estimate is limited, ○○○ ⊕ Very low quality: We have very low confidence in the effect estimate							

### Risk of bias assessment in the included studies

Study quality was assessed separately by 2 researchers (S.B. and M.D.) using the Newcastle-Ottawa scale (NOS) for all observational studies (cross-sectional, case-control, and cohort studies) (33). The scale NOS has 8 stages, which includes 3 domains: selection, comparison, and relating to type of outcome (cohort studies) or exposure (case-control studies). A score of 0 or 1 was assigned for each criterion, depending on whether the benchmark was satisfactorily met. Each study was assigned a point scale of 9 stars, with ratings of 5-9 stars representing high quality, and ratings of 0-4 stars representing low quality. Any discrepancies in the NOS rating of the studies were resolved through discussions among the authors. The quality assessment outcomes for all the included studies are presented in (Table II).

**Table 2:** Quality assessment based on the Newcastle-Ottawa Scale of studies included in this meta-analysis


	**Selection**				**Comparability**	**Exposure**			
**Author, yr (Ref) **	**An adequate definition of case**	**Representativeness of the case**	**Selection of controls**	**Definition of controls**	**Control for an important factor**	**Assessment of exposure**	**The same method of ascertainment for cases and controls**	**Non-response rate**	**Score**
	**Mehmet ** ***et al.*** **,** **2022 (27)**	★	★	★	★	★★	–	★		–	7
**Ascar ** ***et al.*** **,** **2021 (28)**	★	–	★	★	★	–	★	–	5
**Ali ** ***et al.*** **,** **2018 (22)**	★	★	★	★	★	–	★	–	6
**Senturk ** ***et al.*** **,** **2018 (29)**	★	★	★	★	★	–	★	–	6
**Celik ** ***et al.*** **,** **2018 (30)**	★	–	★	★	★	–	★	–	5
**Mierzwicka ** ***et al.*** **,** **2018 (31)**	★	★	★	★	★★	–	★	–	7
**Bu ** ***et al.*** **,** **2012 (32)**	★	★	★	★	★	–	★	–	6
**Celik ** ***et al.*** **,** **2011 (17) **	★	★	★	★	★★	–	★	–	7

### Statistical analysis

Standardized mean difference (SMD) and 95% confidence interval (CI) were used to estimate circulating preptin levels in PCOS cases compared with controls (34, 35). The I^2^ and Cochran's Q test were used to assess heterogeneity between studies; heterogeneity was considered statistically significant (p < 0.05 or I^2^ > 50%) and a random effects model was applied (36, 37).

Publication bias was assessed using Egger's and Begg's tests (38, 39). A p-value of 0.05 was used to determine significant publication bias. Sources of heterogeneity were identified by subgroup analysis and meta-regression. Sensitivity analysis was also performed to calculate the impact of each study by removing a single study. Statistical data analysis was performed using comprehensive meta-analysis v3.7z software.

## 3. Results

### Characteristics of the included studies

**Figure 1:**
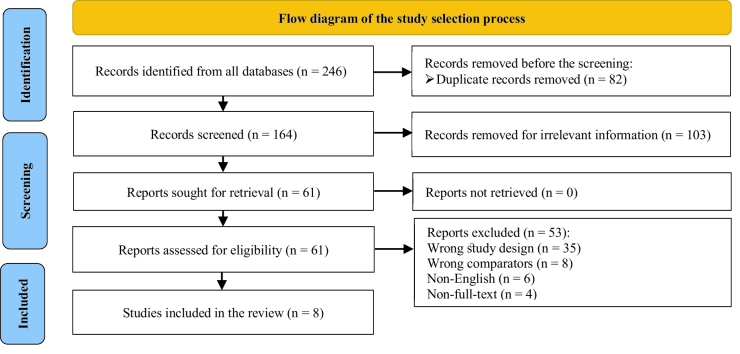
Flow diagram of study selection adjusted by prisma.

The search strategy identified 246 articles in the database, but 82 duplicate records were removed before the screening. A total of 103 articles were excluded after reviewing the titles and abstracts. Afterward, 61 potentially relevant articles were evaluated by a full-text review. Among these articles, 43 studies were excluded because they had the wrong study design and comparison group, and 10 were excluded for other reasons. Thus, 8 studies (involving 582 participants) met our selection criteria (Figure 1).

### Association and comparisons details

In 8 studies with a sample size of 582, preptin levels were examined in PCOS participants compared with the control group. Subgroup analysis and meta-regression analysis were also performed to reveal the source of heterogeneity based on body mass index, age, HOMA-IR, geographic region, and year of the studies. This extensive evaluation has led to an improved understanding of the relationship between preptin levels and PCOS and provides valuable insights into the potential factors that contribute to the observed heterogeneity.

### Relationship between serum preptin levels and PCOS 

#### Meta-analysis 

**Figure 2:**
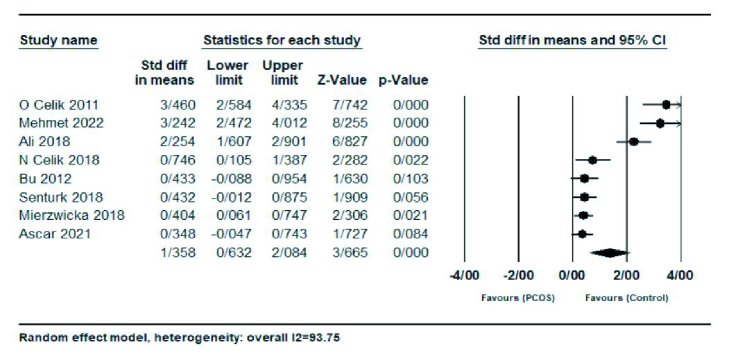
The forest plots comparing serum preptin levels between PCOS and control groups.

Result showed a significantly higher preptin level in the PCOS group compared with control group (SMD = 1.35; 95% CI: 0.63-2.08; p < 0.001) and there was significant heterogeneity between studies ( ${\text{I}}^{2}$ = 93.75%; p < 0.001) (Figure 2).

####  Subgroup analysis

**Figure 3:**
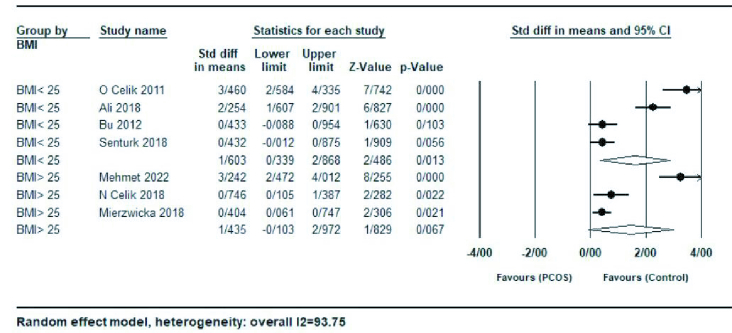
Forest plot of body mass index ≥ 25 kg/m^2^ and body mass index ≤ 25 kg/m^2^ in subgroup analysis.

Subgroup analysis based on BMI, age, and HOMA-IR for each study were also performed. In a subgroup analysis based on studies with a mean BMI ≥ 25 kg/m^2^ or ≤ 25 kg/m^2^, women with BMI > 25 kg/m^2^ (SMD = 1.43; 95% CI: -0.10-2.97; p = 0.06), had lower serum preptin levels compared with the group with BMI < 25 kg/m^2^ (SMD = 1.60; 95% CI: 0.33-2.86; p = 0.01) (Figure 3).

**Figure 4:**
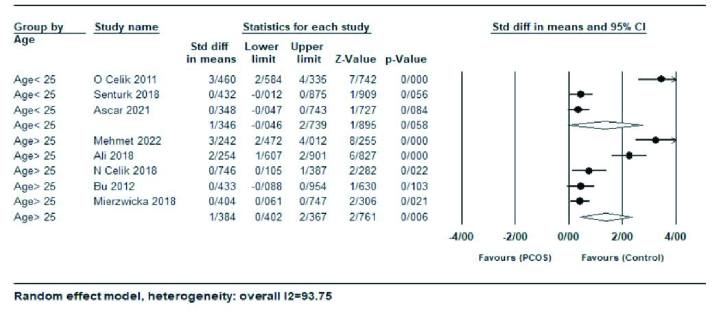
Forest plot of age ≥ 25 yr and age ≤ 25 yr in subgroup analysis.

In a subgroup analysis by studies with mean age ≥ 25 yr or ≤ 25 yr, no significant difference was observed between women with age > 25 yr (SMD = 1.38; 95% CI: 0.40-2.36; p < 0.001) and women with age < 25 yr (SMD = 1.34; 95% CI: -0.04-2.73; p = 0.058) (Figure 4).

**Figure 5:**
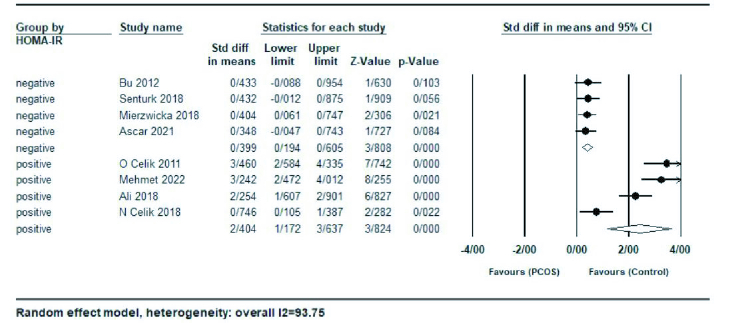
Forest plot of positive/negative HOMA-IR in subgroup analysis.

In a subgroup analysis based on studies with a mean HOMA-IR ≥ 3 (positive for IR) or ≤ 3 (negative for IR), women with HOMA-IR > 3 (SMD = 2.40; 95% CI: 1.17-3.63; p < 0.001), had significantly higher serum preptin levels compared to the group with HOMA-IR < 3 (SMD = 0.39; 95% CI: 0.19-0.60; p < 0.001) (Figure 5).

#### Meta-regression analysis

**Figure 6:**
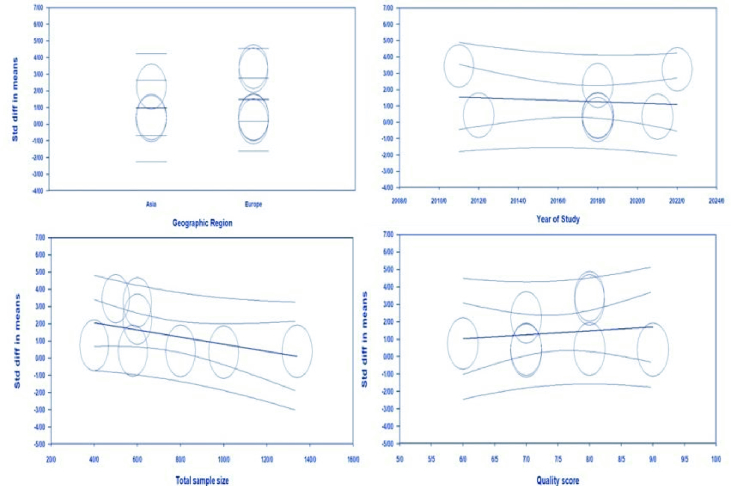
Meta-regression of serum preptin levels in women with PCOS.

The meta-regression for SMD of preptin level based on geographic region (meta-regression coefficient: 0.489; 95% CI: -1.19 to 2.17; p = 0.56), year of studies (meta-regression coefficient: -0.04; 95% CI: -0.26 to 0.17, p = 0.71), sample size (meta-regression coefficient: -0.020; 95% CI: -0.04 to 0.00, p = 0.97), quality score (meta-regression coefficient: 0.224; 95% CI: -0.71 to 1.16, p = 0.64) was not significant (Figure 6).

### Publication bias and sensitivity analysis

**Figure 7:**
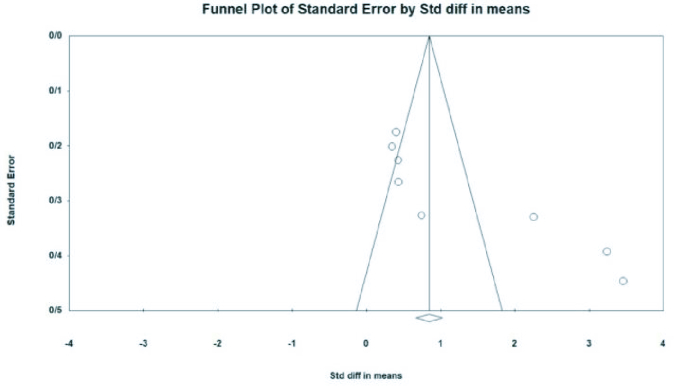
Funnel plot of standard error by standard differences in the means of serum preptin level.

There is substantial evidence of publication bias with respect to the SMD of preptin levels. This bias was identified through both Begg's test (p < 0.001) and Egger's test (p < 0.001) (Figure 7).

However, it is essential to note that the Cochrane handbook for systematic reviews of interventions states that publication bias may be unreliable if fewer than 10 studies were conducted (40). We also performed a sensitivity analysis to confirm the stability and reliability of our outcome. The overall effect size did not change significantly when the individual studies were removed, indicating the reliability of the analysis.

## 4. Discussion

PCOS is a current endocrinal disorder. This syndrome results in infertility, IR, obesity, cardiovascular problems, and other health problems (41). Preptin is an oligopeptide secreted by pancreatic β-cells that has emerged as a potential new biomarker for diagnosing of PCOS because of its role in promoting insulin secretion (42, 43).

Through a meta-analysis of 8 studies conducted between 2011 and 2022, with a total sample size of 582 participants, our findings suggest a significant positive association between high preptin levels and PCOS. These findings provide compelling evidence for the potential role of preptin in the pathogenesis of PCOS, suggesting that preptin may be a key biomarker for identifying and monitoring the progression of this common endocrine disorder. In a previous study, it has shown an increase in serum preptin levels in participants with PCOS compared to the control group (17). Also, in other studies conducted, a significant correlation was suggested between preptin levels in women with PCOS (22). These findings contradict the results of prior reports (32), in which no significant relationship was found between preptin levels and PCOS status.

In this meta-analysis, the value of I^2^ = 93.75% indicates a large heterogeneity between studies; therefore, subgroup analysis and meta-regression were done to determine the cause of heterogeneity. Subgroup analysis was performed based on age, BMI, and HOMA-IR ratio. No significant association was observed between preptin levels and BMI in healthy women compared to PCOS participants, which agrees with previous findings (29, 31). Also, the meta-analysis results based on age showed no significant difference between preptin levels in the PCOS and control groups. The HOMA-IR ratio was considered as a factor for the heterogeneity of the effect size in the analysis of subgroups; according to the results of a subgroup analysis based on the HOMA-IR ratio, a positive correlation was observed between increased preptin levels in women with PCOS and an increased HOMA-IR ratio. In addition, meta-regression analysis was performed to investigate potential factors that may contribute to heterogeneity. The results indicated that the SMD of serum preptin levels were not significantly associated with geographic region, publication year, sample size, or quality score. These findings suggest that the effect of preptin on PCOS is consistent across different regions and study periods and is independent of study size and quality. However, other factors such as age, BMI, and insulin resistance may contribute to the observed heterogeneity, and further investigation is warranted to elucidate these factors.

Although previous studies have presented inconsistent findings regarding the link between PCOS and serum preptin levels, our study is the inaugural meta-analysis to elucidate the association between circulating preptin levels and PCOS. It is imperative to acknowledge and consider the inherent limitations of this meta-analysis during the interpretation of its results. These include the reality that the number of studies is insufficient due to the novelty of the topic. However, further studies with a larger statistical population could support our findings. In addition, the data collected for this research were based on English-language articles, and it was not possible to access non-English-language articles, abstracts, and dissertations. Finally, it should be noted that the results of this study require additional research and should be evaluated with caution.

## 5. Conclusion

Our meta-analysis revealed an association between serum preptin levels and PCOS participants, suggesting that preptin could be used as a novel biomarker for PCOS. Moreover, this association was influenced by IR.

##  Conflict of Interest

The authors declare no conflict of interest.
